# Continuity of care for all? Associations between migration background and personal continuity among persons aged 50 and older in Dutch primary care: a registry-based observational study

**DOI:** 10.1093/fampra/cmaf111

**Published:** 2026-01-22

**Authors:** Bianca T Strooij, Marije T te Winkel, Sharon Remmelzwaal, Pauline Slottje, Petra J M Elders, Karlijn J Joling, Irene G M van Valkengoed, Hein P J van Hout, Marieke T Blom, Otto R Maarsingh

**Affiliations:** General Practice, Amsterdam UMC, Amsterdam 1081 BT, the Netherlands; Health Behaviours and Chronic Diseases, Amsterdam Public Health, Amsterdam 1081 BT, the Netherlands; Aging and Later Life, Amsterdam Public Health, Amsterdam 1081 BT, the Netherlands; General Practice, Amsterdam UMC, Amsterdam 1081 BT, the Netherlands; Aging and Later Life, Amsterdam Public Health, Amsterdam 1081 BT, the Netherlands; Aging and Later Life, Amsterdam Public Health, Amsterdam 1081 BT, the Netherlands; Epidemiology and Data Sciences, Amsterdam UMC, Amsterdam 1081 BT, the Netherlands; General Practice, Academic Network of General Practice (ANHA), Amsterdam UMC, Amsterdam 1081 BT, the Netherlands; General Practice, Amsterdam UMC, Amsterdam 1081 BT, the Netherlands; Health Behaviours and Chronic Diseases, Amsterdam Public Health, Amsterdam 1081 BT, the Netherlands; Aging and Later Life, Amsterdam Public Health, Amsterdam 1081 BT, the Netherlands; Medicine for Older People, Amsterdam UMC, Amsterdam 1081 BT, the Netherlands; Public and Occupational Health, Amsterdam UMC, Amsterdam 1081 BT, the Netherlands; General Practice, Amsterdam UMC, Amsterdam 1081 BT, the Netherlands; Aging and Later Life, Amsterdam Public Health, Amsterdam 1081 BT, the Netherlands; General Practice, Amsterdam UMC, Amsterdam 1081 BT, the Netherlands; Health Behaviours and Chronic Diseases, Amsterdam Public Health, Amsterdam 1081 BT, the Netherlands; General Practice, Amsterdam UMC, Amsterdam 1081 BT, the Netherlands; Aging and Later Life, Amsterdam Public Health, Amsterdam 1081 BT, the Netherlands

**Keywords:** continuity of care, primary care, general practice, migration background, diversity, equity of care

## Abstract

**Background:**

Continuity of care (CoC) is linked to better outcomes. Particularly, older adults and those with chronic conditions, like type 2 diabetes (T2D) and dementia, may benefit from CoC. Individuals with a migration background (MB) face challenges in accessing adequate healthcare. Our aim was to study associations between MB and personal continuity of general practitioner (GP) care among older adults, and in subgroups with T2D and dementia.

**Methods:**

Observational cohort study (2013–8) based on electronic records from 48 Dutch general practices linked to data from Statistics Netherlands. We specifically compared adults who migrated to the Netherlands to those without MB. The Herfindahl–Hirschman Index (HHI; low/medium/high) was used to measure CoC. We used multilevel ordinal regression to estimate associations between MB and CoC, adjusted for follow-up time/age/gender/comorbidity/income/practice.

**Results:**

46 663 individuals aged ≥50 years were included: 72.9% with no MB, 5.7% with Surinamese, 4.3% Moroccan, 2.7% Turkish, 5.1% European, and 9.3% other MB. Compared with those without MB, persons with a Moroccan MB had lower odds of having moderate or high CoC [odds ratio (OR) 0.81, 95% CI 0.74–0.89], and persons with a European MB had higher odds of having moderate or high CoC (OR 1.16, 95% CI 1.07–1.26). Persons with a Moroccan MB in the T2D subgroup had lower odds of having moderate or high CoC (OR 0.75, 95% CI 0.64–0.89). No differences were found in the dementia subgroup.

**Conclusions:**

This study reveals inequalities in personal continuity of GP care by MB in the Netherlands. Interventions to improve CoC should actively incorporate MB groups to promote equitable CoC.

KEY MESSAGES

- Persons with a Moroccan migration background (MB) have lower odds of continuity.
- Persons with a European MB have higher odds of continuity.
- Persons with a Moroccan MB and type 2 diabetes have lower odds of continuity.
- Interventions to improve continuity should incorporate MB.

## Introduction

Continuity of care (CoC) is a core value of primary care. One aspect of CoC is personal continuity, referring to care over time by the same general practitioner (GP), leading to understanding of the patient's context and mutual trust between patient and GP [1, 2]. Personal continuity is still highly valued by both patients and care providers [3], and connected to various important care outcomes. High CoC has been associated with reduced emergency care use [4, 5], hospital admissions [5–7] and healthcare costs [8, 9]. Additionally, high CoC is linked to better medication prescribing and adherence [10–12], improved patient satisfaction [7], and reduced mortality [5, 13, 14].

Older persons and persons with chronic conditions, such as type 2 diabetes (T2D) and dementia, are shown to especially prefer high CoC [15, 16], and benefit from it [12, 17–21]. Other groups of patients that may specifically benefit from high CoC are persons with a migration background (MB), including both those who migrated to the Netherlands and children of migrants. Persons with an MB in Europe are less likely to age in good health [22], with higher prevalence and earlier onset of chronic conditions, including T2D and dementia [23–26], compared to European host populations. In addition, persons with an MB face multiple challenges in receiving adequate care, such as language and communication barriers [27] and unmet healthcare needs [28], especially among older adults and first-generation migrants [23].

Research on personal continuity of GP care for persons with an MB is scarce. In the 2017 NHS GP Patient Survey, respondents from Indian, Pakistani and Bangladeshi ethnic groups answered less often that they were likely to see a preferred GP compared with British and Northern Irish respondents (17–25% versus 38%) [17]. These results were confirmed by Stafford *et al.*, who found that belonging to Bangladeshi, Pakistani, Black African, Black Caribbean, or other Black ethnic groups was associated with lower CoC compared to White ethnic groups in England [29]. However, no such data are available for ethnic minorities that are prevalent in other European countries, such as the Netherlands.

The aim of this study was to investigate the associations between having an MB and personal continuity of GP care among older adults in the Netherlands, specifically comparing continuity in adults who migrated to the Netherlands to those without an MB. We explore this further in subgroups with T2D and dementia. Both T2D and dementia are common chronic diseases in the older population and are primarily managed in general practice. These diseases demand high caregiving intensity, where CoC might be of extra importance. We expect to find lower CoC among persons with an MB compared to persons without an MB, with the largest differences found in the subgroups with T2D and dementia, because barriers in access to appropriate care have been described in these patient groups [30, 31].

## Methods

### Design and data resources

We performed an observational cohort study based on routine care data from electronic GP information systems from general practices associated with the Academic Network of General Practice (ANHA) at Amsterdam University Medical Center. In the Netherlands, general practices act as primary point for medical care during office hours and all citizens are required to be registered in a practice [32]. Access to this care is ensured for all inhabitants by national health coverage. The ANHA structurally collects anonymized data from the electronic health records (EHR) of general practices in the wider Amsterdam area. The selected EHR data were linked on person-level, through a trusted third party, to the Municipal Personal Records Database managed by Statistics Netherlands, the governmental organization responsible for processing statistical data in the Netherlands. The combined anonymised data were made accessible to the researchers in a secured environment provided by Statistics Netherlands under strict privacy conditions.

### Study cohort

In total, 48 general practices associated with ANHA were included, located in the cities Amsterdam and Haarlem. The selected EHR data covered a 6-year observation period (2013–8), representing 269 478 patients. We included persons who were registered at a particular practice for ≥1 year; and had ≥5 contacts with this practice, including ≥2 with a GP during the observation period. We excluded persons younger than 50 years old, because of our focus on older adults. In order to gain insight into potential CoC differences related to having a personal migration history, we excluded individuals born in the Netherlands with one or both parents born abroad (children of migrants). Additionally, we investigated two subgroups: (1) persons with dementia, based on a recorded diagnosis with International Classification of Primary Care (ICPC) [33] code P70 and (2) persons with T2D, based on a recorded diagnosis ICPC-code T90.02, at start of follow-up (timeline shown in Appendix A).

### Determinant: migration background

MB was determined based on country of birth of the individual and both parents. In the Netherlands, 18% of the population has a first-generation MB, with approximately 12% originating from outside Europe [34]. The largest non-European migrant groups are from Türkiye, Morocco, and Surinam. Persons from Türkiye and Morocco were recruited to come to the Netherlands in the 1970s to work, were often low-educated and non-Dutch speaking. Persons from Surinam mostly immigrated around the independence of former Dutch colony Surinam in 1975, are generally higher educated than the Turkish and Moroccan groups, and have good language proficiency [35]. Other MBs were grouped in “other European background” and “other background”, due to small group sizes if further split into individual backgrounds. The reference group was persons without MB, defined as the person as well as both parents being born in the Netherlands.

### Outcome measure: Personal continuity

Personal continuity was determined during the follow-up time, which was from the first GP consultation until the last consultation during the observation period. All contacts with a GP (face-to-face, telephone, online, or home visit) were included, and for each contact it was known whether this was registered by the same GP. The Herfindahl–Hirschman Index (HHI) was calculated per patient as measure of personal continuity [36], with a maximum value of 1.0, meaning optimal continuity. The HHI reflects the extent to which a patient's GP contacts are concentrated among GPs (calculation and example shown in Appendix B).

### Statistical analyses

We described baseline characteristics with mean with standard deviation (SD) or median with interquartile range (IQR) for continuous variables, and absolute and relative frequencies for categorical variables. The HHI was not normally distributed, therefore the outcome was categorized into tertiles of low, moderate, or high personal continuity, as no clinically relevant cut-off values have been previously defined in the literature. We used multilevel ordinal regression models to evaluate associations and calculate odds ratios (ORs), comparing moderate and high CoC groups with the low CoC group. A multilevel approach was used to allow for clustering of patients within practices, fitting models with the practice as random effect and random slope, using maximum likelihood estimation. All models were computed with persons without MB as the reference group, and adjusted for follow-up time and days since registration with the practice. Multiple models were fitted to explore the effect of literature-selected covariates on the found associations: (1) age and sex, (2) additionally comorbidity (number of chronic diseases based on ICPC-1 coding by GPs; Appendix C), and (3) additionally income (yearly disposable household income, standardized to adjust for household size, derived from the Dutch tax authorities database). For the subgroups with T2D or dementia, the variables time since T2D or dementia diagnosis were added in Models 2 and 3. Additionally, interaction terms for sex, age (median), and income (tertiles) with the determinant were included in the fully adjusted models.

Multiple sensitivity analyses were performed to improve the robustness of this study. First, we calculated two other CoC indices, the Usual Provider of Care (UPC) [37] and the Bice–Boxerman Index (BBI) [38], to use as outcome measures and repeated all analyses (Appendix B). Second, we dichotomized all outcome measures and repeated the analyses using multilevel logistic regression models. For data cleaning and analyses, we used R Statistical Software (v4.3.2; R Core Team 2023), and the ordinal R package (Christensen 2023). A *P*-value of <0.05 was considered statistically significant.

### Ethical considerations

The Medical Ethics Committee of Amsterdam UMC (protocol no. VUmc 2015-260 and Amsterdam UMC 2023.0624) has declared that the Medical Research Involving Human Subjects Act does not apply to this study, because anonymized data from Statistics Netherlands and the ANHA database were used. The ANHA database contains EHR data from all patients registered in participation practices, except for those who object to their data being used for scientific research purposes.

## Results

### Study sample

**Figure 1:**
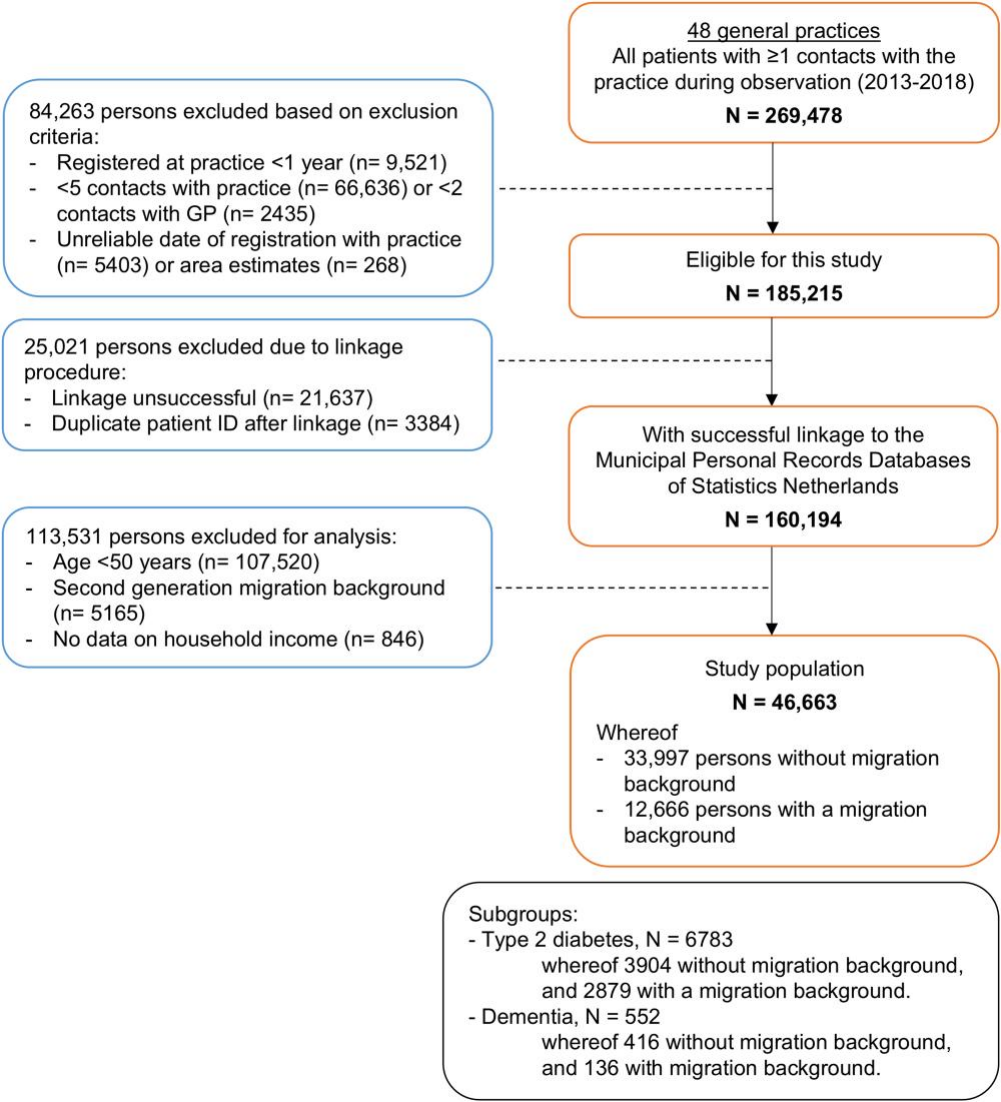
Flowchart of included study population.

A total of 46 663 persons were included in the study, whereof 12 666 with a first-generation MB and 33 997 without an MB (Fig. 1). The subgroups consisted of 6783 persons with T2D (14.5%), and 552 persons with dementia (1.2%). As shown in Table 1, persons with an MB were younger and more often had a lower income, compared to persons without an MB. In addition, persons with an MB had a shorter registration time with the practice, were more often diagnosed with T2D and with cardiovascular diseases than persons without MB. Appendices D and E show the baseline characteristics of the T2D and dementia subgroups.

**Table 1:** Baseline characteristics of study sample, in total and stratified by migration background.

Origin	Netherlands	Turkey	Morocco	Surinam	Europe	Other	Total
	(N = 33 997)	(N = 1272)	(N = 2021)	(N = 2670)	(N = 2366)	(N = 4337)	(N = 46 663)
	72.9%	2.7%	4.3%	5.7%	5.1%	9.3%	
**Follow-up time (years)** Median [Q1, Q3]	4.41 [2.58, 5.41]	5.02 [3.62, 5.67]	5.01 [3.44, 5.65]	4.88 [2.98, 5.59]	4.45 [2.56, 5.45]	4.43 [2.52, 5.42]	4.50 [2.63, 5.45]
**Sex** Female, N (%)	18 847 (55.4)	644 (50.6)	909 (45.0)	1648 (61.7)	1419 (60.0)	2225 (51.3)	25 692 (55.1)
**Age (years)** Median [Q1, Q3]	64.9 [57.1, 73.3]	58.3 [53.2, 66.4]	59.6 [53.9, 67.8]	59.9 [54.6, 68.1]	62.1 [55.6, 70.4]	59.9 [54.5, 67.2]	63.5 [56.2, 72.0]
**Age, in groups based on median** >median, N (%)	18 484 (54.4)	426 (33.5)	745 (36.9)	994 (37.2)	1074 (45.4)	1608 (37.1)	23 331 (50.0)
**Age, in groups based on 65 years** 65 years or older, N (%)	16 840 (49.5)	364 (28.6)	648 (32.1)	877 (32.8)	970 (41.0)	1375 (31.7)	21 074 (45.2)
**Disposable household income per year in tertiles,** N (%)							
Low	9311 (27.4)	765 (60.1)	1327 (65.7)	1216 (45.5)	974 (41.2)	1968 (45.4)	15 561 (33.3)
Intermediate	11 840 (34.8)	343 (27.0)	527 (26.1)	895 (33.5)	694 (29.3)	1249 (28.8)	15 548 (33.3)
High	12 846 (37.8)	164 (12.9)	167 (8.3)	559 (20.9)	698 (29.5)	1120 (25.8)	15 554 (33.3)
**Time of registration in the practice (years)** Median [Q1, Q3]	13.9 [4.45, 20.2]	12.0 [6.03, 17.3]	11.4 [5.54, 17.6]	11.0 [3.14, 17.7]	10.8 [1.93, 18.0]	9.62 [1.51, 16.8]	13.1 [3.95, 19.6]
**Number of GP contacts during follow-up** Median [Q1, Q3]	16.0 [8.00, 29.0]	20.0 [11.0, 33.0]	20.0 [11.0, 33.0]	20.0 [10.0, 36.0]	17.0 [9.00, 30.0]	15.0 [8.00, 27.0]	16.0 [9.00, 29.0]
**Number of GP contacts, per year** Median [Q1, Q3]	4.60 [2.78, 7.73]	4.71 [3.08, 7.32]	4.85 [3.16, 7.37]	5.22 [3.24, 8.70]	4.85 [2.99, 8.15]	4.47 [2.76, 7.44]	4.64 [2.83, 7.77]
**Number of chronic diseases** ^a^ Median [Q1, Q3]	1 [0, 2]	1 [0, 2]	1 [0, 2]	1 [0, 2]	1 [0, 2]	1 [0, 2]	1 [0, 2]
**Chronic diseases** N (%)							
Cardiovascular	5660 (16.6)	238 (18.7)	201 (9.9)	416 (15.6)	293 (12.4)	564 (13.0)	7372 (15.8)
Diabetes type 2	3904 (11.5)	407 (32.0)	726 (35.9)	684 (25.6)	313 (13.2)	749 (17.3)	6783 (14.5)
Cancer	5486 (16.1)	60 (4.7)	111 (5.5)	211 (7.9)	286 (12.1)	365 (8.4)	6519 (14.0)
Respiratory	4361 (12.8)	205 (16.1)	259 (12.8)	298 (11.2)	244 (10.3)	480 (11.1)	5847 (12.5)
Neurological (incl dementia)	1121 (3.3)	30 (2.4)	45 (2.2)	71 (2.7)	63 (2.7)	88 (2.0)	1418 (3.0)
Mental	5120 (15.1)	322 (25.3)	337 (16.7)	420 (15.7)	358 (15.1)	518 (11.9)	7075 (15.2)
***Practice characteristics***							
**Number of listed patients** Median [Q1, Q3]	3490 [2700, 6780]	4060 [3120, 7850]	5030 [3280, 7850]	5130 [3230, 7850]	4220 [2840, 6780]	5030 [2840, 6850]	3650 [2750, 6780]
**Number of listed patients, categorical**							
<2500 patients	6964 (20.5)	185 (14.5)	166 (8.2)	222 (8.3)	337 (14.2)	563 (13.0)	8437 (18.1)
2500–4000 patients	12 151 (35.7)	414 (32.5)	641 (31.7)	858 (32.1)	804 (34.0)	1314 (30.3)	16 182 (34.7)
>4000 patients	14 882 (43.8)	673 (52.9)	1214 (60.1)	1590 (59.6)	1225 (51.8)	2460 (56.7)	22 044 (47.2)
**Number of staff** Median [Q1, Q3]	45.0 [27.0, 60.0]	56.0 [37.3, 75.0]	52.0 [33.0, 69.0]	48.0 [33.0, 63.0]	42.0 [27.0, 60.0]	47.0 [32.0, 60.0]	45.0 [28.0, 63.0]
**Number of staff, excluding GPs** Median [Q1, Q3]	32.0 [21.0, 49.0]	46.0 [28.5, 58.0]	33.0 [23.0, 57.0]	38.0 [23.0, 50.0]	31.0 [20.0, 49.0]	33.0 [23.0, 49.0]	33.0 [21.0, 49.0]
**Number of usual GPs** Median [Q1, Q3]	5.00 [3.00, 7.00]	6.00 [3.00, 8.00]	6.00 [4.00, 8.00]	6.00 [4.00, 7.00]	6.00 [3.00, 7.00]	6.00 [3.00, 7.00]	5.00 [3.00, 7.00]
**Usual GP (years in the practice)** Mean (SD)	4.37 (1.01)	4.10 (1.18)	4.28 (1.14)	4.56 (0.907)	4.48 (1.01)	4.45 (1.04)	4.39 (1.02)
**Number of usual GPs working for >5 years in the practice** Mean (SD)	2.89 (1.85)	2.76 (1.83)	3.13 (1.94)	3.47 (1.87)	3.19 (1.91)	3.24 (1.92)	2.97 (1.87)
**Usual GP (days per year)** Mean (SD)	189 (45.4)	190 (38.8)	191 (36.6)	195 (39.5)	189 (42.1)	187 (41.1)	189 (44.0)
**Number of locum GPs** Median [Q1, Q3]	5.00 [2.00, 8.00]	4.00 [3.00, 13.0]	3.00 [2.00, 8.00]	4.00 [2.00, 8.00]	4.00 [1.00, 7.00]	4.00 [2.00, 7.00]	4.00 [2.00, 7.00]
**Locum GP (% of consults)** ^b^ Median [Q1, Q3]	10.1 [4.2, 17.6]	17.0 [7.6, 21.2]	16.4 [6.8, 21.2]	14.1 [5.3, 21.1]	10.8 [4.2, 18.6]	11.6 [4.2, 20.7]	10.8 [4.2, 18.8]
**Training practice,** N (%)	25 387 (74.7)	878 (69.0)	1351 (66.8)	2115 (79.2)	1695 (71.6)	3178 (73.3)	34 604 (74.2)
***CoC outcome measure***							
**HerfindahlHirschman Index (HHI)**							
Mean (SD)	0.576 (0.213)	0.534 (0.221)	0.513 (0.221)	0.550 (0.214)	0.580 (0.218)	0.559 (0.218)	0.569 (0.215)
**HHI tertiles, n (%)**							
Low (0–0.451)	10 864 (32.0)	489 (38.4)	886 (43.8)	1005 (37.6)	757 (32.0)	1556 (35.9)	
Moderate (0.451–0.643)	11 545 (34.0)	408 (32.1)	614 (30.4)	832 (31.2)	762 (32.2)	1393 (32.1)	
High (0.643–1)	11 588 (34.1)	375 (29.5)	521 (25.8)	833 (31.2)	847 (35.8)	1388 (32.0)	

GP, general practitioner; T2D, type 2 diabetes; SD, standard deviation; IQR, interquartile range; CoC, continuity of care.

^a^Based on comorbidity list, see Appendix C.

^b^Percentage of patient contacts of a practice that are performed by locum GPs.

Generally, persons with an MB were more often registered in larger practices with a higher number of listed patients, a higher number of staff members and a higher number of usual GPs (Table 1). By usual GP we mean a GP who usually works at this practice, self-employed or in salaried service with another self-employed GP. Unlike Kajaria-Montag's “regular doctor” [39], it does not refer to the GP who had the most consultations with a patient over a certain period of time. Also, persons with an MB were more often registered in practices with a higher percentage of patient contacts that are performed by locum GPs, compared to persons without an MB (Table 1).

### Associations of migration background with personal continuity

**Figure 2:**
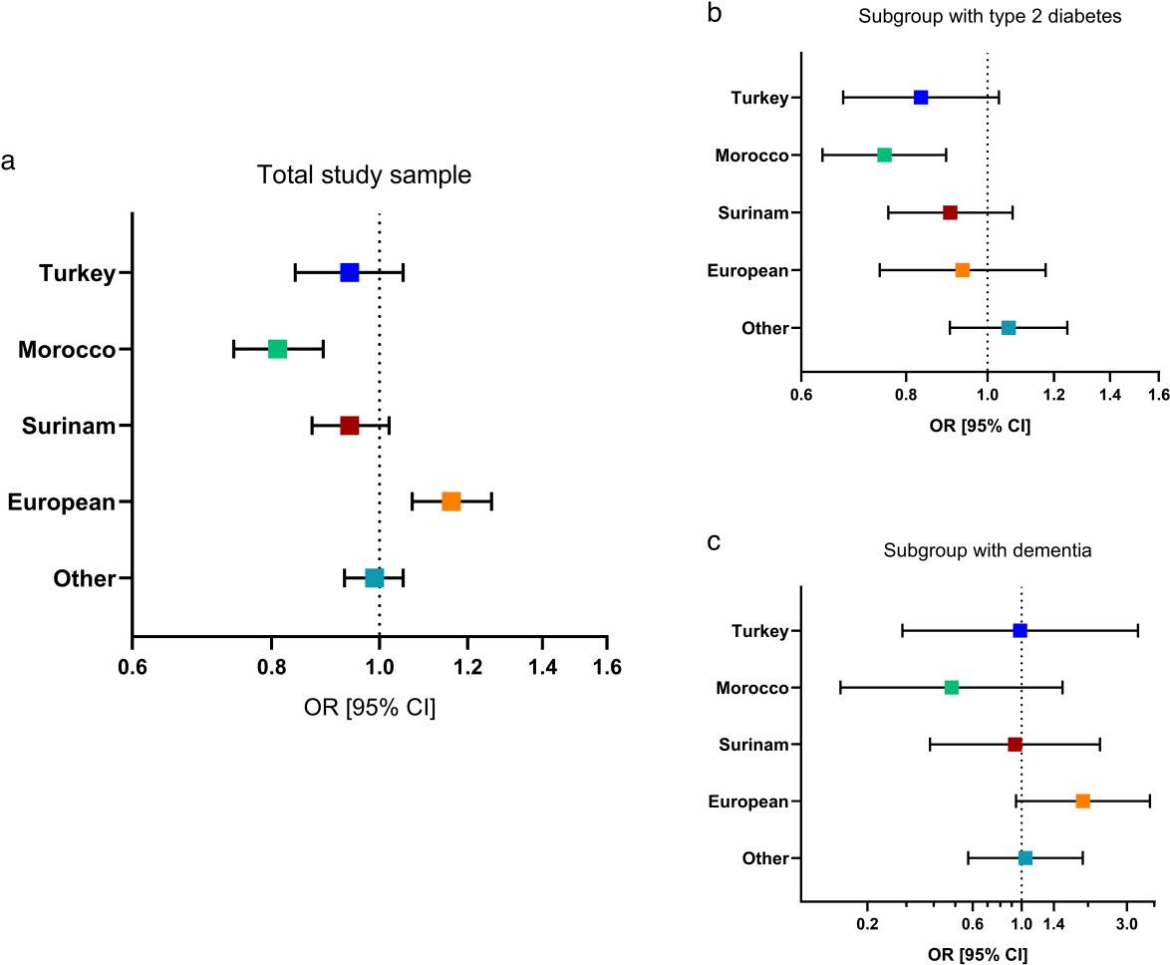
Associations of migration background with personal continuity of GP care (CoC). Data from the Netherlands (2013–8), showing odds ratios (OR) for having moderate or high CoC versus low CoC, with 95% confidence interval (95% CI) for (a) the total study sample (N = 46 663); (b) persons with type 2 diabetes (N = 6783); and (c) persons with dementia (N = 552). Persons without a migration background are the reference group, i.e. individual and both parents born in the Netherlands, based on multilevel ordinal regression models, with adjustment for follow-up time, time of registration at the practice, particular practice, age, registered sex, comorbidity and income.

Mean HHI was highest for persons with a European MB (0.58; SD 0.22) and for persons without an MB (0.58, SD 0.21). The lowest HHI was seen among persons born in Morocco (0.51; SD 0.22). Distribution showed great variability of CoC within all groups. For most groups, the crude ordinal regression analyses showed a negative association between MB and CoC captured by the HHI (Table 2). Only persons with a European MB had higher odds of having moderate or high versus low CoC, compared to persons without an MB (OR 1.13, 95% CI 1.04–1.22). The adjusted analyses all showed associations in the same directions as these crude analyses, with the associations between a Moroccan MB and CoC (OR 0.81, 95% CI 0.74–0.89), and a European MB and CoC (OR 1.16, 95% CI 1.07–1.26) being statistically significant in the fully adjusted model (Fig. 2). The results of the sensitivity analyses, both with other CoC measures (i.e. UPC and BBI) and with dichotomized outcomes, were in line with these findings (Appendices F, G, and H).

**Table 2:** Associations between migration background and personal continuity of GP care in the Netherlands (2013–8), based on multilevel ordinal regression models.

	N	OR	95% CI		*P*-Value	OR	95% CI		*P*-Value	OR	95% CI		*P*-Value	OR	95% CI		*P*-Value
ORIGIN		CRUDE				MODEL 1				MODEL 2				MODEL 3			
**TURKYE**	1272	0.895	0.801	1.000	0.051	0.916	0.819	1.024	0.122	0.919	0.822	1.028	0.138	0.937	0.837	1.049	0.257
**MOROCCO**	2021	0.778	0.711	0.851	<0.001	0.786	0.718	0.861	<0.001	0.787	0.719	0.862	<0.001	0.807	0.736	0.885	<0.001
**SURINAM**	2670	0.906	0.838	0.979	0.012	0.927	0.857	1.002	0.056	0.928	0.858	1.003	0.060	0.939	0.868	1.016	0.115
**EUROPEAN**	2366	1.129	1.041	1.224	0.003	1.147	1.058	1.243	0.001	1.147	1.058	1.243	0.001	1.157	1.067	1.255	<0.001
**OTHER**	4337	0.956	0.899	1.017	0.153	0.973	0.914	1.036	0.390	0.973	0.914	1.035	0.387	0.986	0.926	1.050	0.653

Herfindahl–Hirschman Index (HHI) as measure of personal continuity (CoC), divided in tertiles. ORs for having moderate or high CoC versus low CoC were calculated with persons without a migration background (MB) as the reference group (N = 33 997). For example, for persons with a Turkish MB, the odds of having moderate or high CoC is x times that of persons without an MB. Crude = adjusted for follow-up time, time of registration at the practice and particular practice; model 1 = additional adjustment for age and sex; model 2 = additional adjustment for comorbidity; model 3 = additional adjustment for income.

OR, odds ratio; 95% CI, 95% confidence interval.

### Interaction with sociodemographic variables

We observed no difference in associations between males/females or younger/older adults based on the interaction terms. We did find income-related differences. The analyses of the income-stratified sample showed that, among persons with a high income, having a Turkish MB is associated with lower odds of having intermediate or high CoC (OR 0.714, 95% CI 0.53–0.96) compared to persons without an MB (Appendix I).

### Subgroups with T2D or dementia diagnosis

In the group with a T2D diagnosis, persons with a Moroccan MB also had lower odds of having moderate or high CoC, compared to persons without MB (OR 0.75, 95% CI 0.64–0.89). However, we found no differences in CoC between persons with a European MB and persons without MB in the T2D group. The subgroup with a dementia diagnosis was small. We observed estimates in the same direction as in the main analyses, but no statistically significant associations were found (Appendix J).

## Conclusions

The aim of this study was to investigate possible associations between MB and personal continuity of GP care among older adults in the Netherlands, and to explore this further in subgroups with T2D and dementia. Our findings show that having a Moroccan MB was associated with lower odds of having moderate or high CoC, compared to having no MB, both in the total study population and among persons with a T2D diagnosis. Furthermore, persons with a European MB were found to have higher odds of having moderate or high CoC, compared to persons without MB. These associations remain after adjustment for age, sex, comorbidity and income, and are consistent for different CoC indices.

### Comparison with existing literature

A Danish cohort study found that ethnic minority patients were more likely to experience discontinuity of care in general practice, compared to the host population [40]. In England, Stafford *et al.* showed that five out of ten ethnic minority groups had lower CoC compared with White ethnic patients in primary care [29]. Whilst we focused on different groups of backgrounds, both these and our study concluded that differences in ethnicity or place of birth influence CoC by GPs, irrespective of variations in comorbidity and (socio)economic status. With all the known benefits of higher personal CoC, this might be a worrisome indication of inequality in primary care, possibly resulting in more emergency care use, hospital admissions, inappropriate prescribing and higher healthcare costs in MB groups with lower CoC [4–8, 10].

### Possible mechanisms

In our study, persons with a Moroccan MB had lower CoC compared to persons without MB. Several potential mechanisms may underlie this observed association. One possible explanation might be difficulties with the Dutch language, which could limit patients' ability to request a specific GP. It could also hinder an accurate assessment of urgency, leading to the earliest available appointment with any GP, instead of the regular GP. However, if language barriers were the sole explanation for the observed differences, we would also expect to see lower CoC among the Turkish MB group, not only among individuals with a high income. Particularly given that older adults from both Morocco and Türkiye in the Netherlands demonstrate comparable Dutch language proficiency [41].

Trust may serve as another potential explanation. A lack of trust in a particular GP could lead patients to switch providers, thereby decreasing CoC. Existing literature indicates that patients with an MB often struggle with trust in healthcare professionals in the country of settlement, due to different expectations of the healthcare system [42–44], discrepancy in communication styles between GP and patient [45, 46], and negative experiences such as discrimination [44, 47]. Persons with a Moroccan MB may have lower trust in the GP, compared to persons with another MB or no MB. However, low trust in the Dutch healthcare system has also been described for persons with a Turkish MB in the Netherlands [43]. Additionally, several studies with Dutch-Turkish citizens showed that persons who frequently utilize healthcare in Türkiye, do so because their perceived healthcare needs were not being met in the Netherlands [43, 48]. Unmet (perceived) healthcare needs could lead to medical “shopping”, and switching between GPs, resulting in lower CoC [49]. This may concern mainly persons with a high income, because they have the financial means to pay for healthcare abroad, therefore possibly explaining the lower CoC found among Turkish MB group with a high income.

Several practice characteristics are shown to be negatively associated with personal continuity, such as a large practice size, high number of usual GPs, and many consults by locum GPs [50–52]. In our descriptive analyses, these characteristics seem to differ between persons with and without an MB in our sample, and may therefore partly explain the found differences in CoC. We did however use a multilevel approach to allow for clustering of patients within GP practices, and in this way adjusted for individual differences between practices. Additionally, we know that persons with an MB more often live in socio-economically deprived areas [53], and these areas generally face a higher turnover of GPs [54]. This could result in an increased reliance on temporary staff, which has been identified as a possible explanatory factor in declining CoC [55]. However, this does not explain why the Moroccan MB group exclusively has lower CoC, compared to persons without MB.

### Migration background and ethnicity

The present study concentrated on first-generation MB based on country of birth rather than ethnicity, which has a conceptual overlap but may not fully capture the relevant heterogeneity. Moreover, it is important to acknowledge diversity within the groups with an MB. A risk of studying associations based on MB is generalizing results to all persons with an MB and creating stereotypical beliefs among healthcare professionals [56]. However, persons with a shared MB can differ considerably on religious, geographical, and linguistic characteristics, possibly influencing healthcare use and outcomes. Person-centered care should remain the norm.

### Limitations

Some limitations should be considered while interpreting the results of this study. This study focused on personal continuity, and does not consider other forms of CoC, such as team continuity or informational continuity [57]. Additionally, there is no consensus in literature (yet) on how to best measure personal continuity [36–38, 58]. Various measures, including the HHI, UPC, and BBI, are used interchangeably. Previous studies have shown high correlations between these measures [36]. As the HHI incorporates both frequency and dispersion of patients’ contacts across healthcare providers, we considered this outcome measure to be the most appropriate answer our research question. We used three different CoC indices as outcome measure, which increased the robustness of this study. It can however be debated whether indices are the most accurate representation of CoC. Other proposed measures include Patient Reported Experience Measures (PREMs), such as the NCQ [59], or time of registration at the practice to emphasize CoC as a proxy for the longitudinal doctor–patient relationship [5]. Furthermore, we only used consultations with GPs to calculate the CoC indices. In the Netherlands, other care professionals such as practice nurses are an important part of the primary care team, especially in T2D care. Practice nurses are trained to do protocolized periodic checkups—including structural history taking, physical examination, additional testing, lifestyle advice and medication prescription—and to proactively contact patients over time. Therefore, practice nurses may also positively contribute to personal continuity regardless of whether the patient sees the same GP in GP contacts [60]. It would be informative to include these care relationships in further CoC research. In the analyses, we adjusted for factors known to be associated with personal continuity [50, 52]: age, sex, comorbidity, income, and particular practice, time of registration at that practice and follow-up time. However, we were limited by the variables available in the EHR and administrative databases and by the capabilities of our statistical models. For instance, the variable “number of GP contacts (during follow-up)” could not be added to the models as it is part of the HHI calculation and would cause computational problems. Fortunately, baseline differences in number of GP contacts were small when calculated per year of follow-up.

### Conclusion

The findings in the present study shows inequalities in personal continuity of GP care based on MB in the Netherlands. Older adults with a Moroccan MB were less likely to have intermediate or high CoC compared to persons without an MB. This disparity is concerning because lower CoC has been consistently associated with numerous unfavorable care outcomes. Recognizing these differences can help raise awareness among GPs that care disparities exist across different population groups. Interventions to improve CoC in primary care should actively incorporate MB groups to promote equitable CoC.

## Supplementary Material

cmaf111_Supplementary_Data

## Data Availability

The data underlying this article cannot be shared publicly due to privacy regulations of Statistics Netherlands. Reasonable requests for conditional reuse of the data can be directed to the corresponding author; authorization from Statistics Netherlands is required.
