# Supplementary material for: Continuity of care for all? Associations between migration background and personal continuity among persons aged 50 and older in Dutch primary care: a registry-based observational study
Source: Fam Pract. 2026 Jan 22;43(1):cmaf111. doi: 10.1093/fampra/cmaf111 (PMC12823272; doi:10.1093/fampra/cmaf111)
Supplement: cmaf111_Supplementary_Data [file cmaf111_supplementary_data.pdf]

# Supplementary materials

*With manuscript:*

# Continuity of GP care for all? Associations between migration background and personal continuity of GP care in a registry-based observational study in the Netherlands

**B.T. Strooij, MD[a,b,c]; M. te Winkel, MD[a,c]; S. Remmelzwaal, PhD[c,d]; P. Slottje, PhD[e]; Prof P.J.M. Elders, PhD, MD[a,b]; K.J. Joling, PhD[c,f]; I.G.M. van Valkengoed, PhD[g]; Prof H.P.J. van Hout, PhD[a,c]; M.T. Blom, PhD[a,b] ; Prof O.R. Maarsingh, PhD, MD[a,c]**

[a] Amsterdam UMC, General Practice, Amsterdam, the Netherlands
[b] Amsterdam Public Health, Health Behaviours and Chronic Diseases, Amsterdam, the Netherlands
[c] Amsterdam Public Health, Aging and Later Life, Amsterdam, the Netherlands
[d] Amsterdam UMC, Epidemiology and Data Sciences, Amsterdam, the Netherlands
[e] Amsterdam UMC, General Practice, Academic Network of General Practice (ANHA), Amsterdam, the Netherlands
[f] Amsterdam UMC, Medicine for Older People, Amsterdam, the Netherlands
[g] Amsterdam UMC, Public and Occupational Health, Amsterdam, the Netherlands

## Table of contents

| | Page |
|---|---|
| **Appendix A:** Timeline of used variables in study cohort | **2** |
| **Appendix B:** Personal continuity indices | **3** |
| **Appendix C:** Comorbidity list | **4** |
| **Appendix D:** Baseline characteristics of study sample with type 2 diabetes | **5** |
| **Appendix E:** Baseline characteristics of study sample with dementia | **8** |
| **Appendix F:** Three indices of personal continuity, calculated for the total study sample | **11** |
| **Appendix G:** Analyses with other personal continuity indices: UPC and BBI | **12** |
| **Appendix H:** Sensitivity analysis: multilevel logistic regression | **13** |
| **Appendix I:** Results of multilevel ordinal regression analyses, stratified for income | **14** |
| **Appendix J:** Subgroup analyses; persons with T2D or dementia diagnosis | **15** |

**Appendix A: Timeline of used variables in study cohort**

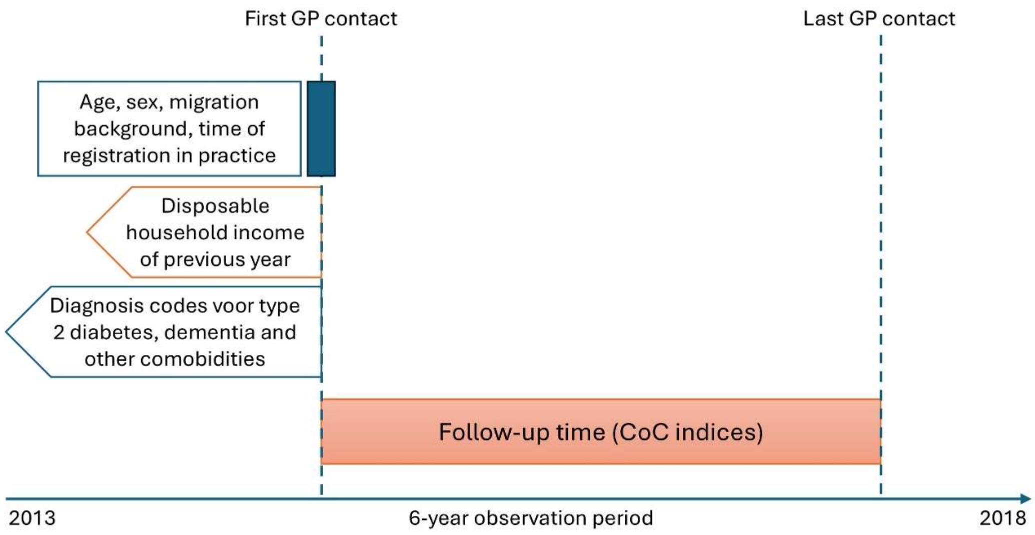


Figure 1. Study diagram depicting observation period, follow-up time of the participants and assessment windows of the used variables. GP = general practitioner; CoC = continuity of care.

**Appendix B: Personal continuity indices used in this study**

Herfindahl-Hirschman Index (HHI):

The HHI reflects the extent to which a patient's contacts are concentrated among general practitioners (GPs). The HHI ranges from 1/N (minimal continuity; every contact with a different GP) to 1.0 (optimal continuity; all GP-contacts were registered with the same GP).

Formula HHI = $\sum_{i=1}^{p} \left(\frac{n_i}{n}\right)^2$

with $p$ = total number of different GPs; $n$ = total number of contacts with GPs; $n_i$ = number of contacts with GP $i$

Usual Provider of Care (UPC):

The UPC expresses the density of GP contacts, considering only a consult with the same GP as continuity of care.

Formula UPC = $\max\left(\frac{n_i}{n}\right)$

with $n$ = total number of contacts with GPs; $n_i$ = number of contacts with GP $i$

Bice-Boxerman Index (BBI):

The BBI shows the dispersion of GP consultations, with higher scores for patients who see fewer GPs.

Formula BBI = $\frac{\left(\sum_{i=1}^{p} n_i^2\right) - n}{n(n-1)}$

with $p$ = total number of different GPs; $n$ = total number of contacts with GPs; $n_i$ = number of contacts with GP $i$

Calculation examples:

Patient X visits his general practice 9 times between 2013 and 2018.
He has 6 contacts with GP A, 2 contacts with GP B and 1 contact with GP C.
Hence, p=3; n=9; n1=6; n2=2; n3=1.

HHI = $(6/9)^2 + (2/9)^2 + (1/9)2 = 0.51$.
UPC = (6/9) = 0.67.
BBI = $((6^2+2^2+1^2)-9) / (9*8) = 0.44$.

**Appendix C: Comorbidity list**

| Chronic disease | ICPC-1 code |
|---|---|
| HIV/AIDS | B90 |
| Cancer | A79, B72, B73, D74, D75, D77, L71, N74, R84, R85, S77, T71, U75, U76, U77, W72, X75, X76, X77, Y77, Y78 |
| Gastric or duodenal ulcer | D85, D86 |
| Chronic enteritis/colitis ulcerosa | D94 |
| Cataract | F92 |
| Hearing disorder | H84, H85 |
| Congenital cardiovascular anomaly | K73 |
| Coronary heart disease | K74, K75, K76 |
| Heart failure | K77 |
| Stroke (including TIA) | K89, K90 |
| Spinal osteoarthritis or spondylosis | L84 |
| Osteoarthritis | L89, L90, L91 |
| Osteoporosis | L95 |
| Congenital neurological anomaly | N85 |
| Multiple sclerosis | N86 |
| Parkinson's disease | N87 |
| Epilepsy | N88 |
| Chronic alcohol abuse | P15 |
| Dementia | P70 |
| Schizophrenia | P72 |
| Anxiety disorder, neurosis, PTSS | P74, P79 |
| Depressive disorder | P76 |
| Mental retardation | P85 |
| Chronic obstructive pulmonary disease | R91, R95 |
| Asthma | R96 |
| Anorexia | T06 |
| Diabetes mellitus | T90 |

Based on: van Oostrom SH, Picavet HS, van Gelder BM, Lemmens LC, Hoeymans N, van Dijk CE, et al. Multimorbidity and comorbidity in the Dutch population - data from general practices. *BMC Public Health*. 2012;12:715.

**Appendix D: Baseline characteristics of study sample with type 2 diabetes in the Netherlands, stratified by migration background**

| Origin | Netherlands | Turkey | Morocco | Surinam | Europe | Other | Total |
|---|---|---|---|---|---|---|---|
| | (N=3904) | (N=407) | (N=726) | (N=684) | (N=313) | (N=749) | (N=6783) |
| | 57.6% | 6.0% | 10.7% | 10.1% | 4.6% | 11.0% | |
| **Follow-up time (years)** Median [Q1, Q3] | 4.63 [2.62, 5.54] | 5.13 [3.76, 5.73] | 5.26 [3.74, 5.70] | 5.14 [2.95, 5.66] | 4.87 [2.55, 5.63] | 4.96 [3.12, 5.58] | 4.87 [2.83, 5.60] |
| **Sex** Female, N (%) | 1865 (47.8%) | 215 (52.8%) | 350 (48.2%) | 429 (62.7%) | 154 (49.2%) | 343 (45.8%) | 3356 (49.5%) |
| **Age (years)** Median [Q1, Q3] | 70.2 [63.4, 78.2] | 62.7 [55.5, 69.7] | 64.0 [57.3, 70.7] | 64.4 [57.8, 72.1] | 67.2 [60.6, 73.4] | 63.7 [57.3, 71.1] | 67.7 [60.2, 75.4] |
| **Age, in groups based on median** >median, N (%) | 2339 (59.9%) | 127 (31.2%) | 267 (36.8%) | 261 (38.2%) | 146 (46.6%) | 251 (33.5%) | 3391 (50.0%) |
| **Age, in groups based on 65 years** 65 years or older, N (%) | 2743 (70.3%) | 165 (40.5%) | 343 (47.2%) | 330 (48.2%) | 188 (60.1%) | 322 (43.0%) | 4091 (60.3%) |
| **Disposable household income per year in tertiles,** N (%) | | | | | | | |
| Low | 871 (22.3%) | 212 (52.1%) | 411 (56.6%) | 299 (43.7%) | 127 (40.6%) | 341 (45.5%) | 2261 (33.3%) |
| Intermediate | 1480 (37.9%) | 112 (27.5%) | 192 (26.4%) | 193 (28.2%) | 90 (28.8%) | 194 (25.9%) | 2261 (33.3%) |
| High | 1553 (39.8%) | 83 (20.4%) | 123 (16.9%) | 192 (28.1%) | 96 (30.7%) | 214 (28.6%) | 2261 (33.3%) |
| **T2D duration (years)** Median [Q1, Q3] | 6.15 [2.96, 10.3] | 6.12 [2.92, 9.82] | 6.70 [3.24, 11.5] | 6.03 [2.86, 11.4] | 4.84 [2.37, 9.68] | 5.39 [2.65, 10.4] | 6.05 [2.91, 10.5] |
| **T2D duration, in groups** | | | | | | | |
| <5 years | 1617 (41.4%) | 172 (42.3%) | 277 (38.2%) | 294 (43.0%) | 158 (50.5%) | 350 (46.7%) | 2868 (42.3%) |
| 5-10 years | 1239 (31.7%) | 137 (33.7%) | 222 (30.6%) | 184 (26.9%) | 81 (25.9%) | 199 (26.6%) | 2062 (30.4%) |
| >10 years | 1048 (26.8%) | 98 (24.1%) | 227 (31.3%) | 206 (30.1%) | 74 (23.6%) | 200 (26.7%) | 1853 (27.3%) |
| **Time of registration in the practice (years)** Median [Q1, Q3] | 15.2 [5.77, 20.4] | 11.6 [5.85, 17.2] | 11.8 [6.31, 18.2] | 9.93 [2.61, 17.9] | 10.7 [3.31, 17.2] | 11.3 [3.02, 17.4] | 13.7 [4.71, 19.8] |

| | | | | | | | |
|---|---|---|---|---|---|---|---|
| **Number of GP contacts during follow-up** Median [Q1, Q3] | 22.0 [12.0, 37.0] | 23.0 [14.0, 36.0] | 23.0 [14.0, 37.0] | 25.0 [14.0, 42.0] | 22.0 [11.0, 36.0] | 20.0 [11.0, 34.0] | 22.0 [12.0, 37.0] |
| **Number of GP contacts, per year** Median [Q1, Q3] | 5.93 [3.61, 10.1] | 5.53 [3.63, 8.00] | 5.38 [3.59, 8.44] | 6.29 [3.78, 10.3] | 5.63 [3.65, 9.30] | 5.22 [3.27, 8.45] | 5.77 [3.60, 9.63] |
| **Number of chronic diseases**[§] Median [Q1, Q3] | 2.00 [1.00, 3.00] | 2.00 [1.00, 3.00] | 2.00 [1.00, 2.75] | 2.00 [1.00, 3.00] | 2.00 [1.00, 3.00] | 2.00 [1.00, 3.00] | 2.00 [1.00, 3.00] |
| **Chronic diseases** N (%) | | | | | | | |
| Cardiovascular | 1278 (32.7%) | 112 (27.5%) | 118 (16.3%) | 195 (28.5%) | 77 (24.6%) | 182 (24.3%) | 1962 (28.9%) |
| Diabetes type 2 | 770 (19.7%) | 25 (6.1%) | 47 (6.5%) | 73 (10.7%) | 41 (13.1%) | 74 (9.9%) | 1030 (15.2%) |
| Cancer | 620 (15.9%) | 75 (18.4%) | 87 (12.0%) | 94 (13.7%) | 38 (12.1%) | 85 (11.3%) | 999 (14.7%) |
| Respiratory | 167 (4.3%) | 17 (4.2%) | 21 (2.9%) | 27 (3.9%) | 12 (3.8%) | 24 (3.2%) | 268 (4.0%) |
| Neurological (incl dementia) | 617 (15.8%) | 84 (20.6%) | 94 (12.9%) | 114 (16.7%) | 51 (16.3%) | 105 (14.0%) | 1065 (15.7%) |
| Mental | 2.00 [1.00, 3.00] | 2.00 [1.00, 3.00] | 2.00 [1.00, 2.75] | 2.00 [1.00, 3.00] | 2.00 [1.00, 3.00] | 2.00 [1.00, 3.00] | 2.00 [1.00, 3.00] |
| | | | | | | | |
| **Practice characteristics** | | | | | | | |
| **Number of listed patients** Median [Q1, Q3] | 3500 [2700, 6780] | 4060 [3230, 7850] | 5030 [3280, 7850] | 5540 [3280, 7850] | 5030 [3090, 6850] | 5130 [3120, 6850] | 4060 [2840, 6780] |
| **Number of listed patients, categorical** | | | | | | | |
| <2500 patients | 771 (19.7%) | 61 (15.0%) | 63 (8.7%) | 51 (7.5%) | 41 (13.1%) | 82 (10.9%) | 1069 (15.8%) |
| 2500-4000 patients | 1396 (35.8%) | 135 (33.2%) | 222 (30.6%) | 225 (32.9%) | 94 (30.0%) | 227 (30.3%) | 2299 (33.9%) |
| >4000 patients | 1737 (44.5%) | 211 (51.8%) | 441 (60.7%) | 408 (59.6%) | 178 (56.9%) | 440 (58.7%) | 3415 (50.3%) |
| **Number of staff** Median [Q1, Q3] | 48.0 [29.0, 75.0] | 56.0 [41.0, 69.0] | 52.0 [33.0, 69.0] | 52.0 [33.0, 69.0] | 48.0 [33.0, 60.0] | 54.0 [33.0, 63.0] | 52.0 [32.0, 69.0] |
| **Number of staff, excluding GPs** Median [Q1, Q3] | 40.0 [23.0, 57.0] | 46.0 [30.0, 58.0] | 40.0 [23.0, 57.0] | 40.0 [26.0, 57.0] | 40.0 [22.0, 49.0] | 44.0 [24.0, 50.0] | 40.0 [23.0, 57.0] |

| | | | | | | | |
|---|---|---|---|---|---|---|---|
| **Number of usual GPs** Median [Q1, Q3] | 5.00 [3.00, 7.00] | 6.00 [4.00, 8.00] | 6.00 [4.00, 8.00] | 6.00 [4.00, 7.00] | 6.00 [3.00, 8.00] | 6.00 [4.00, 8.00] | 6.00 [3.00, 7.00] |
| **Usual GP (years in the practice)** Mean (SD) | 4.31 (1.04) | 4.11 (1.17) | 4.32 (1.13) | 4.60 (0.885) | 4.47 (0.983) | 4.47 (1.05) | 4.35 (1.05) |
| **Number of usual GPs working for >5 years in the practice** Mean (SD) | 2.85 (1.84) | 2.78 (1.81) | 3.17 (1.96) | 3.49 (1.86) | 3.42 (1.95) | 3.30 (1.90) | 3.02 (1.88) |
| **Usual GP (days per year)** Mean (SD) | 189 (45.2) | 190 (37.9) | 191 (36.7) | 195 (39.2) | 189 (40.0) | 185 (38.0) | 190 (42.4) |
| **Number of locum GPs** Median [Q1, Q3] | 5.00 [2.00, 13.0] | 4.00 [3.00, 12.0] | 3.00 [2.00, 7.00] | 4.00 [2.00, 8.00] | 4.00 [2.00, 7.00] | 3.00 [2.00, 7.00] | 4.00 [2.00, 10.0] |
| **Locum GP (% of consults)**† Median [Q1, Q3] | 10.1 [4.23, 18.6] | 17.0 [7.6, 21.2] | 15.1 [5.6, 21.2] | 14.2 [7.7, 21.1] | 11.6 [4.0, 18.6] | 11.6 [4.2, 20.8] | 10.8 [4.2, 20.7] |
| **Training practice,** N (%) | 3008 (77.0%) | 281 (69.0%) | 487 (67.1%) | 536 (78.4%) | 242 (77.3%) | 554 (74.0%) | 5108 (75.3%) |
| | | | | | | | |
| **CoC outcome measure** | | | | | | | |
| **Herfindahl-Hirschman Index (HHI)** | | | | | | | |
| Mean (SD) | 0.583 (0.208) | 0.532 (0.214) | 0.526 (0.222) | 0.555 (0.211) | 0.571 (0.217) | 0.569 (0.220) | 0.569 (0.213) |
| **HHI tertiles, n (%)** | | | | | | | |
| Low (0-0.451) | 1178 (30.2%) | 156 (38.3%) | 310 (42.7%) | 253 (37.0%) | 108 (34.5%) | 256 (34.2%) | |
| Moderate (0.451-0.643) | 1373 (35.2%) | 131 (32.2%) | 219 (30.2%) | 211 (30.8%) | 98 (31.3%) | 229 (30.6%) | |
| High (0.643-1) | 1353 (34.7%) | 120 (29.5%) | 197 (27.1%) | 220 (32.2%) | 107 (34.2%) | 264 (35.2%) | |

T2D = Type 2 diabetes, SD = standard deviation, IQR = interquartile range
§ Based on comorbidity list, see Appendix C. † Percentage of patient contacts of a practice that are performed by locum GPs.

**Appendix E: Baseline characteristics of study sample with dementia in the Netherlands, stratified by migration background**

| Origin | Netherlands | Turkey | Morocco | Surinam | Europe | Other | Total |
|---|---|---|---|---|---|---|---|
| | (N=416) | * | * | * | (N=37) | (N=47) | (N=552) |
| | 75.4% | * | * | * | 6.7% | 8.5% | |
| **Follow-up time (years)** Median [Q1, Q3] | 2.09 [1.07, 3.56] | 1.83 [1.63, 4.46] | 3.31 [2.01, 4.88] | 3.90 [1.94, 5.00] | 2.30 [0.997, 4.61] | 2.42 [1.21, 4.87] | 2.18 [1.09, 3.87] |
| **Sex** Female, N (%) | 253 (60.8%) | <10* | <10* | 19 | 30 (81.1%) | 29 (61.7%) | 342 (62.0%) |
| **Age (years)** Median [Q1, Q3] | 83.1 [74.8, 88.8] | 74.2 [65.2, 78.8] | 69.6 [64.2, 74.8] | 80.4 [70.0, 86.6] | 83.0 [74.0, 89.0] | 82.0 [76.0, 87.4] | 82.2 [73.9, 88.2] |
| **Age, in groups based on median** >median, N (%) | 226 (54.3%) | <10* | <10* | <10* | 19 (51.4%) | 22 (46.8%) | 276 (50.0%) |
| **Age, in groups based on 65 years** 65 years or older, N (%) | 368 (88.5%) | >10* | >10* | >10* | >10* | >10* | 483 (87.5%) |
| **Disposable household income per year in tertiles,** N (%) | | | | | | | |
| Low | 125 (30.0%) | <10* | <10* | 14 | 10 (27.0%) | 17 (36.2%) | 184 (33.3%) |
| Intermediate | 141 (33.9%) | <10* | <10* | <10* | 16 (43.2%) | 14 (29.8%) | 184 (33.3%) |
| High | 150 (36.1%) | <10* | <10* | <10* | 11 (29.7%) | 16 (34.0%) | 184 (33.3%) |
| **Dementia duration (years)** Median [Q1, Q3] | 1.84 [0.60, 4.01] | 1.63 [0.92, 2.40] | 1.67 [1.43, 2.44] | 2.31 [0.91, 3.61] | 2.03 [0.15, 3.52] | 1.23 [0.16, 2.40] | 1.77 [0.59, 3.64] |
| **Dementia duration, in groups** | | | | | | | |
| <5 years | 337 (81.0%) | 12 | 12 | 19 | 33 (89.2%) | >10* | 458 (83.0%) |
| 5-10 years | 63 (15.1%) | <10* | <10* | <10* | <10* | <10* | 75 (13.6%) |
| >10* years | 16 (3.8%) | <10* | <10* | <10* | <10* | <10* | 19 (3.4%) |

| | | | | | | | |
|---|---|---|---|---|---|---|---|
| **Time of registration in the practice (years)** Median [Q1, Q3] | 6.88 [0.21, 18.7] | 8.28 [4.39, 17.4] | 10.8 [4.95, 12.8] | 3.90 [0.08, 15.6] | 9.65 [0.49, 20.3] | 5.78 [0.36, 17.2] | 6.96 [0.22, 18.5] |
| **Number of GP contacts during follow-up** Median [Q1, Q3] | 19.0 [10.0, 34.0] | 16.5 [11.3, 23.0] | 17.0 [8.00, 30.5] | 27.0 [12.5, 39.0] | 17.0 [9.00, 29.0] | 20.0 [7.00, 35.0] | 19.0 [10.0, 34.0] |
| **Number of GP contacts, per year** Median [Q1, Q3] | 10.6 [6.05, 18.4] | 7.29 [3.68, 12.6] | 7.00 [3.61, 12.6] | 8.39 [6.59, 13.1] | 8.56 [5.01, 22.1] | 7.88 [5.78, 11.9] | 9.92 [5.74, 17.9] |
| **Number of chronic diseases**[§] Median [Q1, Q3] | 3.00 [2.00, 4.00] | 5.00 [3.00, 5.75] | 4.00 [2.00, 5.00] | 3.00 [2.00, 4.00] | 3.00 [2.00, 3.00] | 2.00 [1.00, 3.00] | 3.00 [2.00, 4.00] |
| **Chronic diseases** N (%) | | | | | | | |
| Cardiovascular | 148 (35.6%) | 11 | <10* | <10* | 13 (35.1%) | 16 (34.0%) | 203 (36.8%) |
| Diabetes type 2 | 341 (82.0%) | <10* | <10* | 11 | 31 (83.8%) | 32 (68.1%) | 428 (77.5%) |
| Cancer | 89 (21.4%) | <10* | <10* | <10* | <10* | <10* | 103 (18.7%) |
| Respiratory | 48 (11.5%) | <10* | <10* | <10* | <10* | <10* | 60 (10.9%) |
| Neurological (incl dementia) | 370 (88.9%) | 14 | 14 | 20 | 34 (91.9%) | 42 (89.4%) | 494 (89.5%) |
| Mental | 74 (17.8%) | <10* | <10* | <10* | <10* | <10* | 93 (16.8%) |
| | | | | | | | |
| **Practice characteristics** | | | | | | | |
| **Number of listed patients** Median [Q1, Q3] | 3650 [2750, 6780] | 3580 [2460, 5610] | 3650 [3080, 7850] | 5130 [3470, 8280] | 3490 [2750, 6780] | 6560 [3150, 6850] | 4060 [2750, 6850] |
| **Number of listed patients, categorical** | | | | | | | |
| <2500 patients | 72 (17.3%) | <10* | <10* | <10* | <10* | <10* | 89 (16.1%) |
| 2500-4000 patients | 140 (33.7%) | <10* | <10* | <10* | >10* | <10* | 185 (33.5%) |
| >4000 patients | 204 (49.0%) | <10* | <10* | 12 | 17 (45.9%) | 32 (68.1%) | 278 (50.4%) |
| **Number of staff** Median [Q1, Q3] | 43.5 [33.0, 60.0] | 63.5 [44.5, 75.0] | 60.0 [41.0, 73.0] | 42.0 [39.5, 59.0] | 35.0 [18.0, 60.0] | 56.0 [33.0, 60.0] | 45.0 [33.0, 60.0] |

| | | | | | | | |
|---|---|---|---|---|---|---|---|
| **Number of staff, excluding GPs** Median [Q1, Q3] | 32.0 [23.0, 50.0] | 52.0 [31.5, 56.3] | 49.0 [30.0, 58.0] | 33.0 [29.5, 49.5] | 27.0 [14.0, 46.0] | 46.0 [24.5, 49.0] | 32.0 [23.0, 50.0] |
| **Number of usual GPs** Median [Q1, Q3] | 5.50 [3.00, 7.00] | 5.50 [4.25, 6.75] | 6.00 [4.00, 8.00] | 6.00 [4.00, 7.00] | 3.00 [2.00, 9.00] | 6.00 [4.00, 7.50] | 6.00 [3.00, 7.00] |
| **Usual GP (years in the practice)** Mean (SD) | 4.38 (1.00) | 3.82 (1.31) | 4.03 (1.10) | 4.80 (0.592) | 4.77 (0.865) | 4.65 (0.976) | 4.42 (1.00) |
| **Number of usual GPs working for >5 years in the practice** Mean (SD) | 2.88 (1.68) | 2.43 (1.60) | 2.73 (1.83) | 3.83 (1.75) | 3.14 (1.77) | 3.53 (1.69) | 2.98 (1.71) |
| **Usual GP (days per year)** Mean (SD) | 194 (45.5) | 185 (36.9) | 199 (39.8) | 197 (33.3) | 207 (45.1) | 192 (42.8) | 195 (44.5) |
| **Number of locum GPs** Median [Q1, Q3] | 5.00 [2.00, 7.00] | 8.00 [3.00, 16.0] | 4.00 [3.00, 12.5] | 3.00 [2.00, 4.50] | 3.00 [2.00, 5.00] | 3.00 [2.00, 5.00] | 4.00 [2.00, 7.00] |
| **Locum GP (% of consults)**† Median [Q1, Q3] | 10.8 [4.2, 17.6] | 11.2 [7.6, 17.9] | 21.1 [8.5, 21.2] | 14.2 [4.1, 19.8] | 6.8 [4.2, 14.1] | 7.6 [4.2, 15.1] | 10.1 [4.2, 17.6] |
| **Training practice,** N (%) | 311 (74.8%) | <10* | 10 | 21 | 25 (67.6%) | 39 (83.0%) | 415 (75.2%) |
| | | | | | | | |
| **CoC outcome measure** | | | | | | | |
| **Herfindahl-Hirschman Index (HHI)** | | | | | | | |
| Mean (SD) | 0.589 (0.207) | 0.583 (0.217) | 0.486 (0.211) | 0.527 (0.163) | 0.701 (0.214) | 0.600 (0.227) | 0.592 (0.211) |
| **HHI tertiles, n (%)** | | | | | | | |
| Low (0-0.451) | 138 (33.2%) | <10* | <10* | 10 | <10* | 14 (29.8%) | |
| Moderate (0.451-0.643) | 147 (35.3%) | <10* | <10* | <10* | >10* | 18 (38.3%) | |
| High (0.643-1) | 131 (31.5%) | <10* | <10* | <10* | 19 (51.4%) | 15 (31.9%) | |

T2D = Type 2 diabetes, SD = standard deviation, IQR = interquartile range, CoC = continuity of care.
* Exact value censored due to privacy regulations of Statistics Netherlands, to prevent possible group identification of the study sample.
§ Based on comorbidity list, see Appendix C. † Percentage of patient contacts of a practice that are performed by locum GPs.

**Appendix F: Three indices of personal continuity, calculated for the total study population.**

| **Origin** | Netherlands | Turkey | Morocco | Surinam | Europe | Other | Total |
|---|---|---|---|---|---|---|---|
| | (N=33,997) | (N=1272) | (N=2021) | (N=2670) | (N=2366) | (N=4337) | (N=46,663) |
| **Usual Provider of Care (UPC)** | | | | | | | |
| Mean (SD) | 0.686 (0.191) | 0.652 (0.204) | 0.631 (0.207) | 0.667 (0.195) | 0.689 (0.195) | 0.669 (0.198) | 0.680 (0.194) |
| **UPC tertiles, n (%)** | | | | | | | |
| Low (0-0.583) | 11,123 (32.7) | 480 (37.7) | 868 (42.9) | 954 (35.7) | 772 (32.6) | 1556 (35.9) | |
| Moderate (0.583-0.786) | 11,460 (33.7) | 416 (32.7) | 638 (31.6) | 892 (33.4) | 759 (32.1) | 1412 (32.6) | |
| High (0.786-1) | 11,414 (33.6) | 376 (29.6) | 515 (25.5) | 824 (30.9) | 835 (35.3) | 1369 (31.6) | |
| **Herfindahl-Hirschman Index (HHI)** | | | | | | | |
| Mean (SD) | 0.576 (0.213) | 0.534 (0.221) | 0.513 (0.221) | 0.550 (0.214) | 0.580 (0.218) | 0.559 (0.218) | 0.569 (0.215) |
| **HHI tertiles, n (%)** | | | | | | | |
| Low (0-0.451) | 10,864 (32.0) | 489 (38.4) | 886 (43.8) | 1005 (37.6) | 757 (32.0) | 1556 (35.9) | |
| Moderate (0.451-0.643) | 11,545 (34.0) | 408 (32.1) | 614 (30.4) | 832 (31.2) | 762 (32.2) | 1393 (32.1) | |
| High (0.643-1) | 11,588 (34.1) | 375 (29.5) | 521 (25.8) | 833 (31.2) | 847 (35.8) | 1388 (32.0) | |
| **Bice-Boxerman Index (BBI)** | | | | | | | |
| Mean (SD) | 0.533 (0.241) | 0.497 (0.240) | 0.473 (0.241) | 0.512 (0.237) | 0.539 (0.246) | 0.511 (0.247) | 0.526 (0.242) |
| **BBI tertiles, n (%)** | | | | | | | |
| Low (0-0.400) | 11,512 (33.9) | 504 (39.6) | 905 (44.8) | 1000 (37.5) | 794 (33.6) | 1674 (38.6) | |
| Moderate (0.400-0.613) | 10,905 (32.1) | 375 (29.5) | 579 (28.6) | 825 (30.9) | 726 (30.7) | 1311 (30.2) | |
| High (0.613-1) | 11,580 (34.1) | 393 (30.9) | 537 (26.6) | 845 (31.6) | 846 (35.8) | 1352 (31.2) | |

**Appendix G: Analyses with other continuity of care (CoC) indices: Usual Provider of Care (UPC) and Bice-Boxerman Index (BBI)**

We investigated the associations between migration background (MB) and CoC with multilevel ordinal regression analyses, using persons without an MB as the reference group (N=33,997). Odds ratios (OR) and 95% confidence intervals (95%CI) were calculated for having moderate of high CoC, compared to the reference group. E.g. for persons with a Turkish MB, the odds of having moderate or high CoC is … times that of persons without an MB.

| | HHI | OR | 95% CI | | P-VALUE | UPC | OR | 95% CI | | P-VALUE | BBI | OR | 95% CI | | P-VALUE |
|---|---|---|---|---|---|---|---|---|---|---|---|---|---|---|---|
| | | **CRUDE** | | | | | **CRUDE** | | | | | **CRUDE** | | | |
| TURKYE (N=1272) | | 0.895 | 0.801 | 1.000 | 0.051 | | 0.909 | 0.814 | 1.015 | 0.089 | | 0.898 | 0.803 | 1.003 | 0.057 |
| MOROCCO (N=2021) | | 0.778 | 0.711 | 0.851 | <0.001 | | 0.774 | 0.708 | 0.846 | <0.001 | | 0.765 | 0.699 | 0.837 | <0.001 |
| SURINAM (N=2670) | | 0.906 | 0.838 | 0.979 | 0.012 | | 0.924 | 0.856 | 0.998 | 0.045 | | 0.925 | 0.856 | 0.999 | 0.046 |
| EUROPEAN (N=2366) | | 1.129 | 1.041 | 1.224 | 0.003 | | 1.109 | 1.024 | 1.202 | 0.011 | | 1.117 | 1.031 | 1.211 | 0.007 |
| OTHER (N=4337) | | 0.956 | 0.899 | 1.017 | 0.153 | | 0.956 | 0.899 | 1.017 | 0.154 | | 0.907 | 0.852 | 0.964 | 0.002 |
| | | **MODEL 1** | | | | | **MODEL 1** | | | | | **MODEL 1** | | | |
| TURKYE | | 0.916 | 0.819 | 1.024 | 0.122 | | 0.938 | 0.840 | 1.048 | 0.257 | | 0.955 | 0.854 | 1.068 | 0.417 |
| MOROCCO | | 0.786 | 0.718 | 0.861 | <0.001 | | 0.789 | 0.721 | 0.862 | <0.001 | | 0.799 | 0.730 | 0.875 | <0.001 |
| SURINAM | | 0.927 | 0.857 | 1.002 | 0.056 | | 0.95 | 0.879 | 1.026 | 0.188 | | 0.962 | 0.890 | 1.039 | 0.325 |
| EUROPEAN | | 1.147 | 1.058 | 1.243 | 0.001 | | 1.130 | 1.042 | 1.224 | 0.003 | | 1.148 | 1.060 | 1.245 | 0.001 |
| OTHER | | 0.973 | 0.914 | 1.036 | 0.390 | | 0.981 | 0.922 | 1.043 | 0.540 | | 0.952 | 0.895 | 1.013 | 0.123 |
| | | **MODEL 2** | | | | | **MODEL 2** | | | | | **MODEL 2** | | | |
| TURKYE | | 0.919 | 0.822 | 1.028 | 0.138 | | 0.935 | 0.837 | 1.045 | 0.234 | | 0.939 | 0.840 | 1.050 | 0.269 |
| MOROCCO | | 0.787 | 0.719 | 0.862 | <0.001 | | 0.788 | 0.720 | 0.862 | <0.001 | | 0.794 | 0.726 | 0.869 | <0.001 |
| SURINAM | | 0.928 | 0.858 | 1.003 | 0.060 | | 0.949 | 0.878 | 1.025 | 0.181 | | 0.957 | 0.886 | 1.034 | 0.270 |
| EUROPEAN | | 1.147 | 1.058 | 1.243 | 0.001 | | 1.130 | 1.043 | 1.224 | 0.003 | | 1.149 | 1.060 | 1.245 | 0.001 |
| OTHER | | 0.973 | 0.914 | 1.035 | 0.387 | | 0.981 | 0.922 | 1.043 | 0.543 | | 0.953 | 0.896 | 1.014 | 0.128 |
| | | **MODEL 3** | | | | | **MODEL 3** | | | | | **MODEL 3** | | | |
| TURKYE | | 0.937 | 0.837 | 1.049 | 0.257 | | 0.950 | 0.850 | 1.062 | 0.365 | | 0.948 | 0.847 | 1.061 | 0.351 |
| MOROCCO | | 0.807 | 0.736 | 0.885 | <0.001 | | 0.804 | 0.734 | 0.880 | <0.001 | | 0.804 | 0.734 | 0.881 | <0.001 |
| SURINAM | | 0.939 | 0.868 | 1.016 | 0.115 | | 0.958 | 0.887 | 1.035 | 0.277 | | 0.963 | 0.891 | 1.041 | 0.345 |
| EUROPEAN | | 1.157 | 1.067 | 1.255 | <0.001 | | 1.138 | 1.050 | 1.233 | 0.002 | | 1.154 | 1.065 | 1.251 | <0.001 |
| OTHER | | 0.986 | 0.926 | 1.050 | 0.653 | | 0.991 | 0.932 | 1.055 | 0.785 | | 0.959 | 0.901 | 1.021 | 0.189 |

**Appendix H: Sensitivity analysis: multilevel logistic regression**

Outcome measures were dichotomized based on the first tertile (low vs moderate/high personal CoC), and multilevel logistic regression models were made. Crude = adjusted for follow-up time, time of registration in the practice and particular practice; model 1 = additional adjustment for age and sex; model 2 = additional adjustment for comorbidity; model 3 = additional adjustment for income. OR= odds ratio for having moderate or high CoC, compared to the reference group (persons without MB, N=33,997). This yielded similar results as our main analyses (see manuscript).

| | HHI | OR | 95% CI | | P-VALUE | UPC | OR | 95% CI | | P-VALUE | BBI | OR | 95% CI | | P-VALUE |
|---|---|---|---|---|---|---|---|---|---|---|---|---|---|---|---|
| | | **CRUDE** | | | | | **CRUDE** | | | | | **CRUDE** | | | |
| TURKYE (N=1272) | | 0.913 | 0.797 | 1.045 | 0.187 | | 0.908 | 0.796 | 1.036 | 0.151 | | 0.870 | 0.763 | 0.993 | 0.039 |
| MOROCCO (N=2021) | | 0.782 | 0.704 | 0.87 | <0.001 | | 0.775 | 0.699 | 0.859 | <0.001 | | 0.748 | 0.675 | 0.830 | <0.001 |
| SURINAM (N=2670) | | 0.891 | 0.812 | 0.977 | 0.014 | | 0.925 | 0.845 | 1.013 | 0.093 | | 0.922 | 0.842 | 1.009 | 0.076 |
| EUROPEAN (N=2366) | | 1.099 | 0.995 | 1.214 | 0.062 | | 1.075 | 0.976 | 1.183 | 0.143 | | 1.092 | 0.991 | 1.202 | 0.075 |
| OTHER (N=4337) | | 0.937 | 0.870 | 1.01 | 0.089 | | 0.936 | 0.871 | 1.007 | 0.075 | | 0.885 | 0.824 | 0.951 | 0.001 |
| | | **MODEL 1** | | | | | **MODEL 1** | | | | | **MODEL 1** | | | |
| TURKYE | | 0.932 | 0.813 | 1.067 | 0.308 | | 0.943 | 0.827 | 1.076 | 0.386 | | 0.944 | 0.827 | 1.077 | 0.393 |
| MOROCCO | | 0.790 | 0.710 | 0.879 | <0.001 | | 0.794 | 0.716 | 0.881 | <0.001 | | 0.796 | 0.717 | 0.883 | <0.001 |
| SURINAM | | 0.911 | 0.831 | 1.00 | 0.049 | | 0.952 | 0.869 | 1.043 | 0.292 | | 0.968 | 0.884 | 1.060 | 0.478 |
| EUROPEAN | | 1.116 | 1.010 | 1.233 | 0.031 | | 1.095 | 0.995 | 1.206 | 0.064 | | 1.127 | 1.023 | 1.242 | 0.015 |
| OTHER | | 0.953 | 0.884 | 1.028 | 0.212 | | 0.966 | 0.898 | 1.039 | 0.349 | | 0.947 | 0.881 | 1.019 | 0.143 |
| | | **MODEL 2** | | | | | **MODEL 2** | | | | | **MODEL 2** | | | |
| TURKYE | | 0.938 | 0.819 | 1.075 | 0.361 | | 0.937 | 0.821 | 1.069 | 0.334 | | 0.918 | 0.804 | 1.048 | 0.206 |
| MOROCCO | | 0.792 | 0.712 | 0.881 | <0.001 | | 0.793 | 0.715 | 0.879 | <0.001 | | 0.788 | 0.710 | 0.874 | <0.001 |
| SURINAM | | 0.913 | 0.832 | 1.002 | 0.054 | | 0.950 | 0.868 | 1.041 | 0.274 | | 0.960 | 0.877 | 1.051 | 0.381 |
| EUROPEAN | | 1.116 | 1.010 | 1.233 | 0.031 | | 1.096 | 0.995 | 1.207 | 0.063 | | 1.129 | 1.025 | 1.244 | 0.014 |
| OTHER | | 0.953 | 0.884 | 1.027 | 0.21 | | 0.966 | 0.898 | 1.039 | 0.352 | | 0.948 | 0.882 | 1.02 | 0.152 |
| | | **MODEL 3** | | | | | **MODEL 3** | | | | | **MODEL 3** | | | |
| TURKYE | | 0.962 | 0.839 | 1.104 | 0.582 | | 0.952 | 0.833 | 1.087 | 0.464 | | 0.922 | 0.807 | 1.054 | 0.235 |
| MOROCCO | | 0.816 | 0.732 | 0.909 | <0.001 | | 0.807 | 0.727 | 0.896 | <0.001 | | 0.792 | 0.713 | 0.880 | <0.001 |
| SURINAM | | 0.926 | 0.844 | 1.017 | 0.107 | | 0.959 | 0.875 | 1.051 | 0.369 | | 0.962 | 0.878 | 1.054 | 0.408 |
| EUROPEAN | | 1.128 | 1.020 | 1.246 | 0.018 | | 1.103 | 1.001 | 1.215 | 0.047 | | 1.131 | 1.027 | 1.246 | 0.013 |
| OTHER | | 0.968 | 0.897 | 1.044 | 0.400 | | 0.975 | 0.906 | 1.05 | 0.502 | | 0.951 | 0.884 | 1.023 | 0.177 |

**Appendix I: Results of multilevel ordinal regression analyses, stratified for income**

Showing associations between migration background and personal continuity of GP care in the Netherlands (2013-2018), stratified in income-groups. Based on Herfindahl–Hirschman Index (HHI) as a measure of personal continuity, divided in tertiles. Model = adjusted for follow-up time, registration time in the practice, particular practice, age, sex, comorbidity and income.

| HHI | N (%) | P-VALUE INTERACTION TERM | OR | 95% CI | | P-VALUE |
|---|---|---|---|---|---|---|
| **LOW INCOME** | | | | | | |
| NETHERLANDS | 9311 (27.4) | | 1.00 | | | |
| TURKYE | 765 (60.1%) | | 0.923 | 0.794 | 1.074 | 0.301 |
| MOROCCO | 1327 (65.7%) | | 0.805 | 0.715 | 0.906 | <0.001 |
| SURINAM | 1216 (45.5%) | | 0.962 | 0.853 | 1.085 | 0.531 |
| EUROPEAN | 974 (41.2%) | | 1.125 | 0.989 | 1.280 | 0.074 |
| OTHER | 1968 (45.4%) | | 0.966 | 0.875 | 1.065 | 0.483 |
| | | | | | | |
| **INTERMEDIATE INCOME** | | | | | | |
| NETHERLANDS | 11,840 (34.8%) | | 1.00 | | | |
| TURKYE | 343 (27.0%) | 0.594 | 1.048 | 0.848 | 1.295 | 0.666 |
| MOROCCO | 527 (26.1%) | 0.380 | 0.769 | 0.644 | 0.918 | 0.004 |
| SURINAM | 895 (33.5%) | 0.874 | 0.953 | 0.832 | 1.091 | 0.484 |
| EUROPEAN | 694 (29.3%) | 0.900 | 1.184 | 1.021 | 1.373 | 0.025 |
| OTHER | 1249 (28.8%) | 0.587 | 0.973 | 0.866 | 1.093 | 0.642 |
| | | | | | | |
| **HIGH INCOME** | | | | | | |
| NETHERLANDS | 12,846 (37.8%) | | 1.00 | | | |
| TURKYE | 164 (12.9%) | 0.017 | 0.714 | 0.532 | 0.957 | 0.024 |
| MOROCCO | 167 (8.3%) | 0.162 | 0.705 | 0.518 | 0.961 | 0.027 |
| SURINAM | 559 (20.9%) | 0.076 | 0.859 | 0.728 | 1.014 | 0.073 |
| EUROPEAN | 698 (29.5%) | 0.958 | 1.156 | 0.997 | 1.340 | 0.055 |
| OTHER | 1120 (25.8%) | 0.498 | 0.966 | 0.859 | 1.088 | 0.572 |

**Appendix J: Subgroup analyses; persons with T2D or dementia diagnosis**

Associations between migration background (MB) and personal continuity of GP care in the Netherlands (2013-2018). Based on Herfindahl–Hirschman Index (HHI) as a measure of personal continuity, divided in tertiles. We made multilevel ordinal regression models, with persons without MB as the reference group to calculate odds ratios for having moderate or high CoC, compared to the reference group. E.g. for persons with a Turkish MB, the odds of having moderate or high CoC is … times that of persons without MB. Crude = adjusted for follow-up time, time of registration in practice and particular practice; model 1 = additional adjustment for age and sex; model 2 = additional adjustment for comorbidity; model 3 = additional adjustment for income. CoC = continuity of care; OR = odds ratio; 95%CI = 95% confidence interval.

**T2D diagnosis:**

| | N | OR | 95% CI | | P-VALUE | OR | 95% CI | | P-VALUE | OR | 95% CI | | P-VALUE | OR | 95% CI | | P-VALUE |
|---|---|---|---|---|---|---|---|---|---|---|---|---|---|---|---|---|---|
| **ORIGIN** | | **CRUDE** | | | | **MODEL 1** | | | | **MODEL 2** | | | | **MODEL 3** | | | |
| NETHERLANDS | 3904 | 1.00 | | | | 1.00 | | | | 1.00 | | | | 1.00 | | | |
| TURKYE | 407 | 0.804 | 0.653 | 0.990 | 0.040 | 0.806 | 0.653 | 0.996 | 0.046 | 0.817 | 0.661 | 1.009 | 0.060 | 0.833 | 0.673 | 1.032 | 0.094 |
| MOROCCO | 726 | 0.741 | 0.629 | 0.873 | <0.001 | 0.740 | 0.627 | 0.874 | <0.001 | 0.735 | 0.622 | 0.868 | <0.001 | 0.754 | 0.636 | 0.893 | 0.001 |
| SURINAM | 684 | 0.867 | 0.734 | 1.024 | 0.093 | 0.888 | 0.750 | 1.051 | 0.166 | 0.893 | 0.754 | 1.057 | 0.189 | 0.903 | 0.762 | 1.071 | 0.240 |
| EUROPEAN | 313 | 0.925 | 0.738 | 1.160 | 0.500 | 0.928 | 0.740 | 1.164 | 0.518 | 0.924 | 0.737 | 1.159 | 0.495 | 0.934 | 0.744 | 1.173 | 0.559 |
| OTHER | 749 | 1.053 | 0.900 | 1.231 | 0.519 | 1.047 | 0.893 | 1.228 | 0.569 | 1.043 | 0.890 | 1.223 | 0.603 | 1.060 | 0.902 | 1.245 | 0.479 |

**Dementia diagnosis:**

| | N | OR | 95% CI | | P-VALUE | OR | 95% CI | | P-VALUE | OR | 95% CI | | P-VALUE | OR | 95% CI | | P-VALUE |
|---|---|---|---|---|---|---|---|---|---|---|---|---|---|---|---|---|---|
| **ORIGIN** | | **CRUDE** | | | | **MODEL 1** | | | | **MODEL 2** | | | | **MODEL 3** | | | |
| NETHERLANDS | 416 | 1.00 | | | | 1.00 | | | | 1.00 | | | | 1.00 | | | |
| TURKYE | 14 | 0.942 | 0.290 | 3.064 | 0.921 | 0.807 | 0.246 | 2.648 | 0.724 | 0.833 | 0.248 | 2.799 | 0.768 | 0.984 | 0.288 | 3.367 | 0.980 |
| MOROCCO | 15 | 0.477 | 0.158 | 1.446 | 0.191 | 0.397 | 0.129 | 1.223 | 0.108 | 0.422 | 0.134 | 1.326 | 0.140 | 0.481 | 0.151 | 1.533 | 0.216 |
| SURINAM | 23 | 0.772 | 0.325 | 1.833 | 0.557 | 0.851 | 0.354 | 2.049 | 0.719 | 0.845 | 0.351 | 2.035 | 0.708 | 0.933 | 0.384 | 2.263 | 0.878 |
| EUROPEAN | 37 | 1.767 | 0.888 | 3.517 | 0.105 | 1.896 | 0.948 | 3.791 | 0.070 | 1.871 | 0.936 | 3.742 | 0.076 | 1.897 | 0.944 | 3.812 | 0.072 |
| OTHER | 47 | 1.042 | 0.576 | 1.886 | 0.891 | 1.045 | 0.577 | 1.891 | 0.885 | 1.019 | 0.562 | 1.846 | 0.951 | 1.040 | 0.572 | 1.892 | 0.898 |
